# Antimicrobial peptide polymers: no escape to ESKAPE pathogens—a review

**DOI:** 10.1007/s11274-020-02907-1

**Published:** 2020-08-01

**Authors:** Songhita Mukhopadhyay, A. S. Bharath Prasad, Chetan H. Mehta, Usha Y. Nayak

**Affiliations:** 1 Department of Pharmaceutics, Manipal College of Pharmaceutical Sciences, Manipal Academy of Higher Education, Manipal, Karnataka 576104 India; 2 Department of Ageing Research, Manipal School of Life Sciences, Manipal Academy of Higher Education, Manipal, Karnataka 576104 India

**Keywords:** Antimicrobial resistance, *ESKAPE* pathogens, Antimicrobial peptides, Structurally nanoengineered antimicrobial peptide polymers (SNAPPs), Nanostructured antimicrobial peptides

## Abstract

**Abstract:**

Antimicrobial resistance (AMR) is one of the significant clinical challenges and also an emerging area of concern arising from *nosocomial infections of ESKAPE pathogens,* which has been on the rise in both the developed and developing countries alike*.* These pathogens/superbugs can undergo rapid mutagenesis, which helps them to generate resistance against antimicrobials in addition to the patient’s non-adherence to the antibiotic regimen. Sticking to the idea of a ‘one-size-fits-all’ approach has led to the inappropriate administration of antibiotics resulting in augmentation of antimicrobial resistance. Antimicrobial peptides (AMPs) are the natural host defense peptides that have gained attention in the field of AMR, and recently, synthetic AMPs are well studied to overcome the drawbacks of natural counterparts. This review deals with the novel techniques utilizing the bacteriolytic activity of natural AMPs. The effective localization of these peptides onto the negatively charged bacterial surface by using nanocarriers and structurally nanoengineered antimicrobial peptide polymers (SNAPPs) owing to its smaller size and better antimicrobial activity is also described here.

**Graphic abstract:**

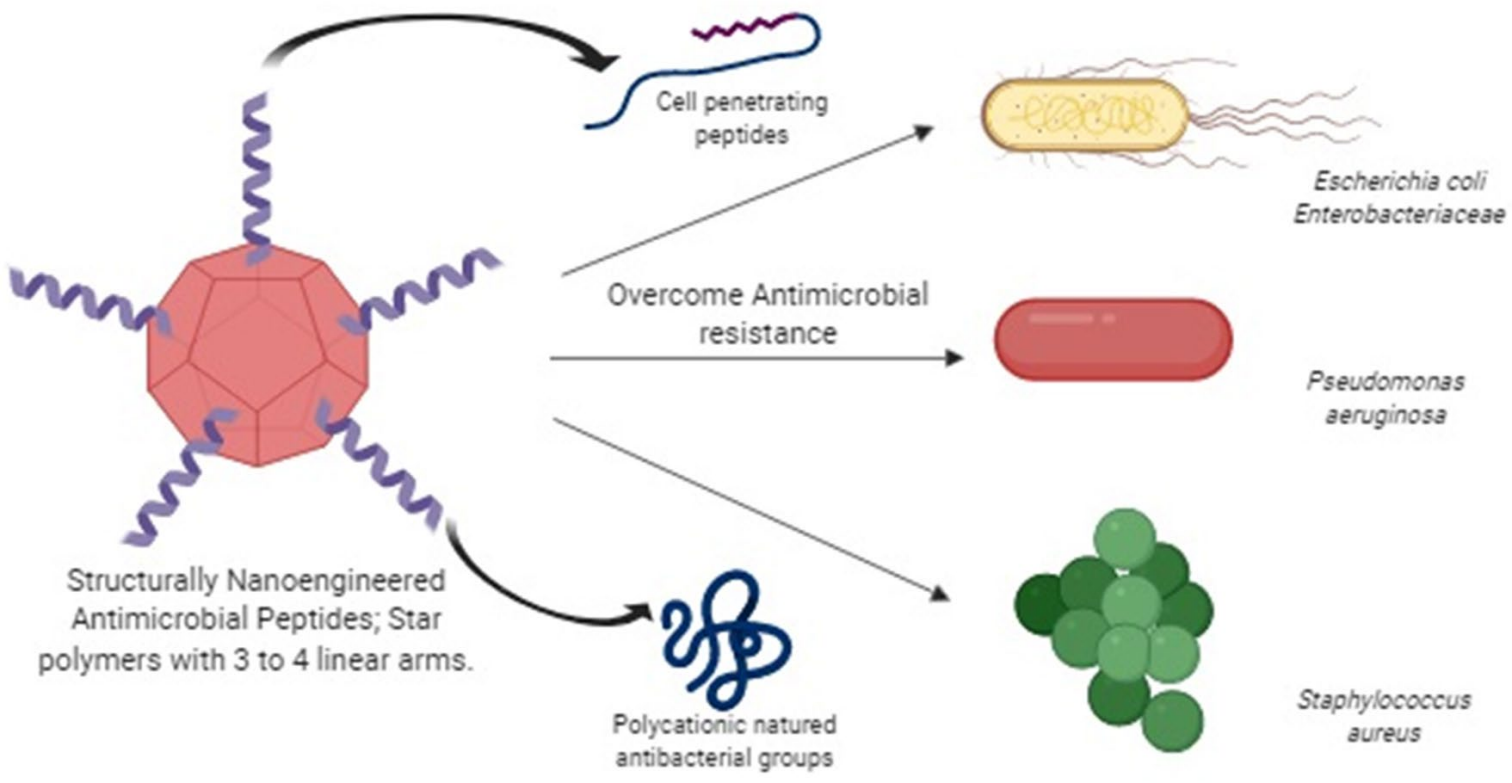

## Introduction

A variety of organisms, including bacteria, fungi, viruses, and parasites, are mainly responsible for causing nosocomial infections. The surveillance studies from the hospital and Infectious Diseases Society of America has designated the group of pathogens responsible for causing nosocomial infections as ESKAPE pathogens. ESKAPE pathogens are a group of bacteria containing both Gram-positive and Gram-negative bacteria, namely, *Enterococcus faecium, Staphylococcus aureus, Klebsiella pneumoniae, Acinetobacter baumannii, Pseudomonas aeruginosa*, and *Enterobacter species*. As per the WHO reports, the mortalities due to drug resistant-strains of ESKAPE bugs such as *Klebsiella pneumoniae*, *Escherichia coli*, *Neisseria gonorrhoeae*, and *Staphylococcus aureus* have increased in the past few years. Analysis of the bacterial genomes has led to the conclusion that there is a shortage of potent antibiotics because around 20,000 potential resistant genes are reported. The USA alone has showed 99,000 deaths, which were associated with the hospital-acquired infections. The two most common hospital-acquired diseases are sepsis, and pneumonia which has caused around 55,000 deaths resulting in both microbial as well as economic burden (Aslam et al. 2018; Dixit et al. 2019). ESKAPE bugs infections have been growing in both the developed and the developing countries alike (Rice 2008).

The first and foremost reason behind the spread of nosocomial infection is the poor hygienic conditions, sanitation, and malnourishment. But, the most challenging part of this epidemic is its treatment. Physicians prescribe an enormous number of combinations of antibiotics without considering its side effects. Even if a disease whose cause may be a narrow spectrum bacterium which can be treated by a single low dose antibiotic regimen, they are being forced to be processed by giving high dose combinations to overcome the antimicrobial resistance (AMR). The concept of antimicrobial resistance comes from the fact of inadequate antibacterial therapy, which deals with treating the bacterial disease with an antibiotic to which the bacteria are not susceptible. This leads to an increased resistance in the circulating bacterial agents in the human body. Inappropriate or overuse of antibiotics may also lead to antimicrobial resistance (Olesen et al. 2018). Among ESKAPE pathogens, methicillin-resistant *Staphylococcus aureus* (MRSA), extended-spectrum β-lactamase producing (ESBL) *Escherichia coli* and vancomycin-resistant Enterococcus (VRE) are commonly seen. Although these agents have gained popularity, they exert resistance against antimicrobial agents in the healthcare set-up. A relationship of resistance between the host-immune responses with the frequency of biofilm formation has already been established (Tenover 2006; Murali et al. 2014). WHO has come out with a global priority list of antibiotic-resistant bacteria which guides in the new antibiotic development (Table 1). Certain pathogens have been grouped as ESKAPE pathogens by the WHO against which new antibiotics are essential. The discovery of novel therapy for the treatment of drug-resistant infections, particularly those caused by ESKAPE pathogens is time-consuming. Hence, antibiotics in conjunctions, synthetic chemicals, phages, antimicrobial peptides (AMPs), nanomaterials, and photodynamic light therapy have been recommended as an alternative method (Mulani et al. 2019; Ma et al. 2020). Out of these measures, AMPs, natural host defense peptides, containing a backbone of amino acids are found to be promising candidates, which can be an alternative to the conventional antibiotics that develop resistance. Although AMPs have shown successful results,they also suffer from various drawbacks such as toxicity, susceptibility to proteolysis, poor pharmacokinetics profile, and many more. The development of nanocarriers or nanomaterials encapsulated with AMPs helps in minimizing the degradation and cytotoxicity with increased efficiency of AMPs at the target site (Brandelli 2012). In addition to nanocarriers, the star polymers and structurally nanoengineered antimicrobial peptide polymers are also used to deliver the AMPs due to its significant advantages and potential therapeutic efficacy (Lam et al. 2016).

**Table 1:** WHO recommended global priority list of antibiotic-resistant bacteria

Priority	Antibiotic-resistant bacteria	Drug to which it is resistant
Priority1: CRITICAL *	*Acinetobacter baumannii*	Carbapenem
	*Pseudomonas aeruginosa*	Carbapenem
	*Enterobacteriaceae* #	Carbapenem, 3rd generation cephalosporins
Priority2: HIGH	*Enterococcus faecium*	Vancomycin
	*Staphylococcus aureus*	Methicillin, Vancomycin intermediate and resistant
	*Helicobacter pylori*	Clarithromycin
	*Campylobacter*	Fluoroquinolones
	*Salmonella *spp.	Fluoroquinolones
	*Neisseria gonorrhoeae*	3rd generation cephalosporins and fluoroquinolone
Priority3: MEDIUM	*Streptococcus pneumoniae*	Penicillin
	*Haemophilus influenzae*	Ampicillin
	*Shigella *spp.	Fluoroquinolone

^*^Mycobacteria (responsible for Tuberculosis) was not included in this priority list as it is already established, and new treatments are coming up; # Enterobacteriaceae consists of the following species: *Klebsiella pneumonia, Escherichia coli, Enterobacter *spp.*, Serratia *spp.*, Proteus *spp.*, Providencia *spp.*, Morganella *spp.

This review deals with the AMPs with its mechanism of action and bacteriolytic activity against combating the ESKAPE crisis. Further, the emphasis is given on the delivery of AMPs using nanocarriers, novel nanostructures such as star polymers, and structurally nanoengineered antimicrobial peptide polymers (SNAPPs). The effective localization of these peptides onto the negatively charged bacterial surface owing to its smaller size which may enhance the antimicrobial activity is also discussed.

## Antimicrobial peptides (AMPs)

AMPs are the peptides produced naturally by the multicellular organisms as the first line of defense against pathogenic microbes during infections. AMPs are amphiphilic in nature with cationic charge and relatively smaller in size (10–50 amino acids). Human have an innate immunity to microbial infections such as lysozyme secreted by the nasal mucosa which acts as a bacteriolytic. These enzymes are polypeptide in nature. Polypeptides are widely being known for their characteristic size that might exist in different conformation such as primary, secondary, tertiary, quaternary which adds to its flexibility, amphiphilic nature, and surface charge, which is complementary to the cell membrane surface charge of the bacteria. Subgroup I anionic peptides contain zinc (Zn) as a cofactor, which is essential for the antimicrobial efficacy, and subgroup II lacks cysteine residue,and forms a α-helical structures in the presence of sodium dodecyl sulfate (Hancock and Sahl 2006). There are different mechanisms by which it hampers or obstructs the resistance development. It targets the cell wall of bacteria by forming electrostatic interactions between the anionic bacterial cell wall membranes with AMPs cationic residues, which shows the bactericidal activity and, in addition to this, the insertion of hydrophilic subunits into the cytoplasmic membrane of bacteria leads to the permeabilization or disruption, thus causing the cell death. AMPs also act by forming the pores on the bacterial cell membrane which causes the death of the bacteria (Kamaruzzaman et al. 2019; Namivandi-Zangeneh et al. 2019). Since then, various classes of AMPs have been identified which are listed out in Table 2 (Ashley et al. 2018).

**Table 2:** Classes of antimicrobial peptide (Ashley et al. 2018)

Class	Peptide	Source
Anionic	Maximin H5 Dermicidin	Amphibians Humans
Linear Cationic α-helical	Cecropins (A) Andropin Moricin Ceratotoxin Melittin	Insects
Enriched with specific amino acid (cationic)	Proline containing abaecin Tryptophan containing indolicidin	Honeybee Cattle
Anionic & cationic containing cysteine with disulfide bonds	2-disulfide bridges (protegrin) 3-disulfide bridges (α-defensins)	Pigs Human (HNP-1*, HNP-2, Cryptidins)
Anionic & cationic peptide fragments	Lactoferricin Casocidin I Bovine α-lactalbumin antimicrobial domain, Haemoglobin, lysozyme, ovalbumin	Lactoferrin Human casein Bovine, human

^*^: Human Neutrophil Peptides

The characteristics of an ideal antimicrobial peptide are listed below: (Brogden 2005):

- Size: approximately 6–59 amino acids chain length and anionic nature
- Sequencing: Should contain basic amino acid residues like arginine/lysine, hydrophobic residues like alanine, leucine, and phenylalanine. The ratio of hydrophobic to a charged concentration should be 1:1 or 2:1.
- Configuration: Preferably α-helical. Some of the AMPs are found in the form of two antiparallel β-sheets (γ-core motif).
- Sufficiently hydrophobic to partition through the cell membrane.
- Amphipathicity, expressed as a hydrophobic moment, summing up all the hydrophobic vector residues in the helical structure that are easy to calculate compared to those peptides with spatial configuration.

### Mechanism of action of AMPs

**Fig. 1:**
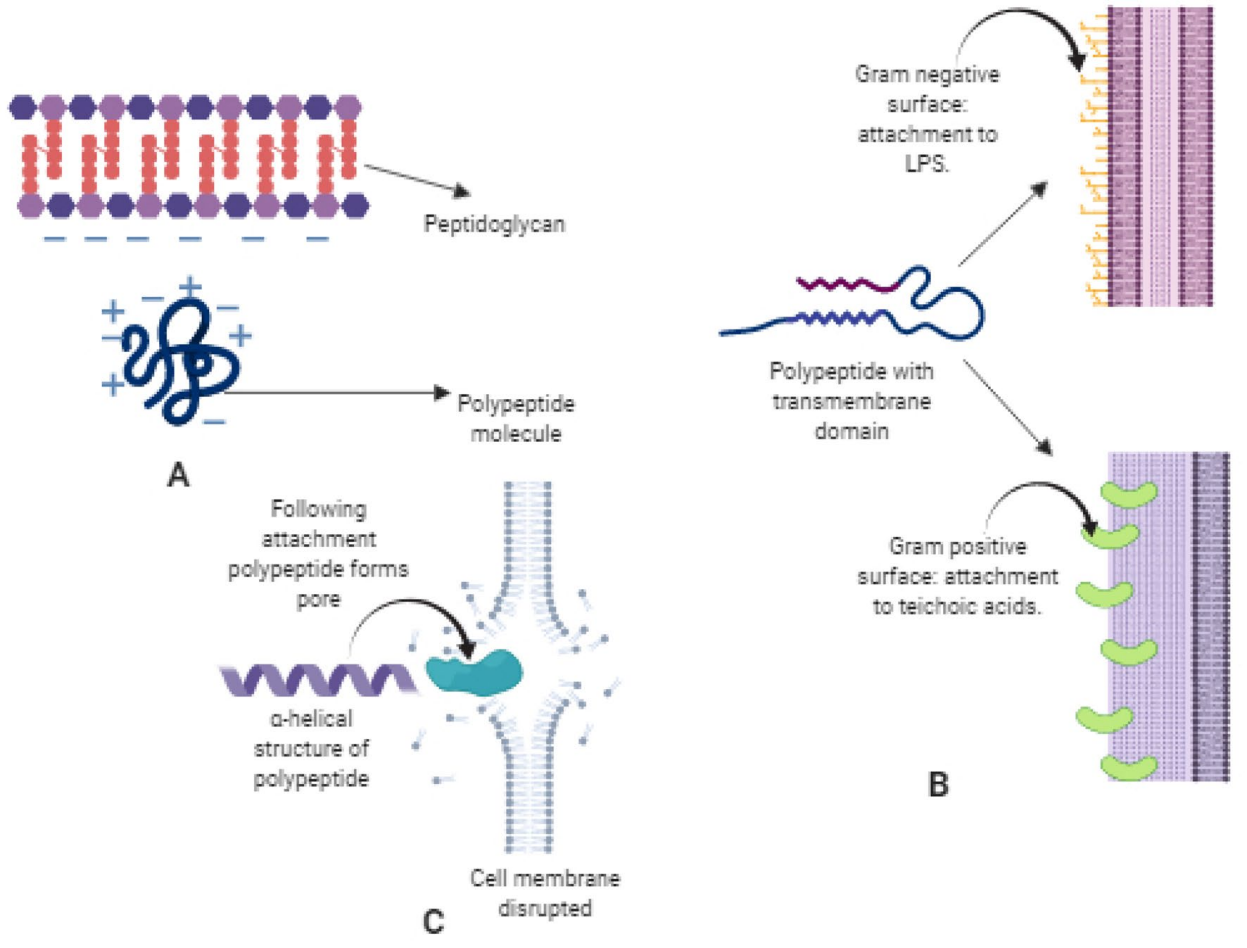
General mechanism of action of antimicrobial peptides; **a** represents attraction step where electrostatic bonding arises between negatively charged peptidoglycan layer of the bacterial cell membrane and amphiphilic polypeptide structure; **b** represents attachment step where the AMPs binds to Lipopolysaccharide (LPS) layer of Gram-negative cell wall and a teichoic acid layer of Gram-positive cell wall; **c** Represents the final peptide insertion step where following attachment the peptide forms a pore and thus disrupts the bacterial cell membrane

**Fig. 2:**
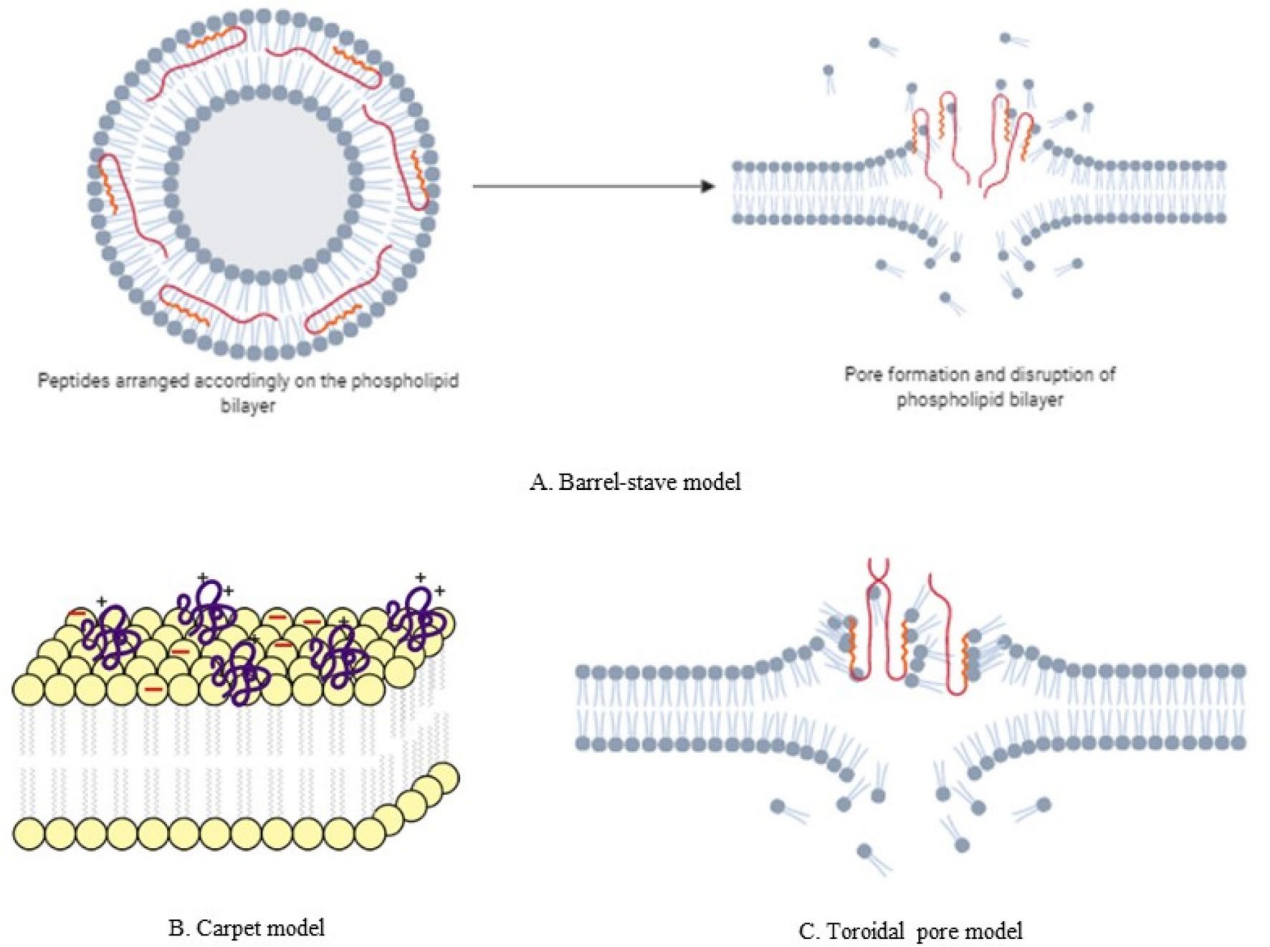
**a** Barrel-stave model of pore formation by AMPs, the hydrophobic region marked by red color aligns itself to the lipophilic part of the phospholipid bilayer, and the hydrophilic part represented by small orange part aligns itself towards the hydrophilic region of the phospholipid bilayer. **b** Carpet model of AMPs induced cell killing. Electrostatic bonding between the negatively charged bacterial cell surface and polypeptides aligns parallel to the cell membrane. **c** Toroidal pore model. AMPs induce the bending of the lipid monolayer in such a way that the polar head groups are both associated with the inserted peptides as well as the lipophilic

Two basic mechanisms of short cationic amphiphilic host defense peptides responsible for the antimicrobial activity are direct cell killing and immunomodulatory action. Several models have been proposed to explain the disruption of the cell membrane caused by the AMPs. Amongst them, the 'Barrel-stave', 'Carpet model', and 'Toroidal-pore' are the major ones (Fig. 1) (Zasloff 2019). AMPs mediated cell killing involves three basic steps namely, attraction, attachment, and peptide insertion. Attraction step involves the basic electrostatic bonding of the charged cationic/anionic peptides and negatively charged units of the bacterial surface. In the attachment step, the peptide must penetrate the whole distance of the polysaccharide bacterial surface and join with the lipopolysaccharide, especially from Gram-negative bacteria or teichoic and lipoteichoic acid from Gram-positive bacteria. The α-helical structures are more effective as they can attach the bacterial membrane at even low peptide/lipid ratios. Following the attachment, any of the above said models are applied for creating a pore in the cell membrane surface, thus disrupting the cell membrane integrity. At the initial stages, I-state in which the peptides are parallel to the lipid surface are formed. As the concentration of peptide increases, it aligns itself perpendicular to the cell membrane. In the 'Barrel-stave' model, the hydrophobic region aligns itself towards the lipidic portion, and the hydrophilic region forms the inside portion of the pore (Fig. 2a). In the 'Carpet model,' the peptides are electrostatically bonded to the negatively charged cell membrane in such a way that it is spread all over (Fig. 2b). Antimicrobial activity is exerted upon the disruption of the cell membrane at higher peptide concentrations. In the 'Toroidal pore' model, the inserted peptides cause bending of the lipidic portions in such a way which gives a structure of pore (Fig. 2c) (Dar et al. 2016).

Apart from the physical disruption as pore formers, AMPs also exert their intracellular killing activity by the metabolic modulators. Activating apoptotic behavior of the bacterial cell by upregulation of autolysins e.g. N-acetylmuramoyl-L-alanine which acts as an autolysin activator, modulators of DNA replication like Buforin II; inhibition of DNA, RNA and protein synthesis by pleurocidin, dermaseptin, HNP-1, HNP-2 and inhibition of enzymatic activity by histatins, drosocin, apidaecin have been the proposed mechanisms of these peptides.

### Peptide-based antimicrobial products

AMPs are made either by non-ribosomal peptide synthesis or ribosomal translation of mRNA. Recently, the ribosomal derived peptides produced by all species, have gained wide therapeutic potential (Mahlapuu et al. 2016). Conventional techniques of solid-phase peptide synthesis methods have not been up to the mark in peptide-based antibiotic development technology. Increasing the spectrum of activity and cost-effectiveness are the two main factors that have been focused upon to achieve a reliable formulation objective. Preparation of the peptide arrays involves the incorporation of smaller peptide fragments on the spot using cellulose sheets and then determining its antimicrobial efficacy. Development of peptidomimetic compounds that contains analogs which block the synthesis of a certain enzyme, can be used to inhibit protease synthesis, which resolves the stability issue. (Dar et al. 2016).

Synthetic approach to synthesize such antimicrobial peptides, however, has led to an excellent development in the field of peptide-based antimicrobials. Nevertheless, even notorious microorganisms like *P. aeruginosa* and *E. coli* have shown the tendency to acquire resistance against such products due to their ability of rapid mutagenesis (Mendelson et al. 2016, 2017). The high osmolarity, flavonoids, phytochemical constituents (methyl glyoxal which is 1,2 dicarbonyl compound), lysine/ arginine side chains are involved in the uptake of these constituents in certain diseases. These components of manuka honey and its importance in combating the antimicrobial resistance of *P. aeruginosa* have been described by Shenoy et al. and Alvarez-Suarez et al. (Shenoy et al. 2012; Alvarez-Suarez et al. 2014).

### Resistance to AMPs

Similar to the conventional and other modified dosage forms of antibiotics, bacteria have shown resistance against these AMPs. The mechanism by which resistance is induced includes the alteration of the bacterial cell surface to release the various proteolytic enzymes which results in the hydrolysis of the peptides, for e.g. *S. aureus* alters the net surface charge towards a less negative by introducing basic groups like D-ala, and *K. pneumoniae* forms a capsular body which limits the penetration of the AMPs. Increasing the fluidity of the outer membrane surface by alteration of the lipid A portion as in *Salmonella *spp., modulation of the outer membrane proteins as in *Yersinia* Kaczmarek *enterocolitica* (Hay et al. 2018), presence of active efflux transporters and presence of proteolytic enzymes like metalloproteinase (aureolysin) in *S. aureus* has resulted in an increased resistance to AMPs. For specific Gram-negative bacteria, especially *Enterobacteriaceae*, they exert resistant mechanisms in the form of adaptation to AMPs, proteolytic degradation of AMPs, and shielding of the bacterial cell surface, thus limiting the penetration of AMPs. PhoPQ, PmrAB, and RcsBCD Phosphorelay system are the signaling pathways controlled by the genes of *Enterobacteriaceae,* which codes for AMPs resistance. Proteolytic enzymes like elastase from *P. aeruginosa* have been found to inactivate AMPs LL-37. Most of the periodontal disease-causing microbes like *Porphyromonas gingivalis* secrete AMPs thus inactivating the enzymes like proteases. Proteases released by the outer membrane site is the primary cause of AMPs degradation in *Enterobacteriaceae *spp. Formation of capsule polysaccharides as in *K. pneumoniae*, complex formation of AMPs with exopolysaccharides as in *P. aeruginosa,* and modification of the O-polysaccharide in the outer membrane site (Gruenheid and Le Moual 2012) are the major steps involved in shielding of the bacterial cell surface against AMPs.

The emergence of resistance to conventional antibiotics by several microorganisms has further augmented the research in the case of AMPs. AMPs have played an important role as a self-defense mechanism as well as penetration enhancer for certain antibiotics (Chowdhury et al. 2018; Fontela et al. 2018). Many novel drug delivery systems were attempted to deliver AMPs and to reduce their resistance. Table 3 provides information about the latest antimicrobial peptides, which are currently under clinical trials (https://clinicaltrials.gov/, NIH).

**Table 3:** Antimicrobial peptides currently under clinical trials ( https://clinicaltrials.gov/, NIH)

Title	ClinicalTrials.gov Identifier	Sponsor	Condition/disease	Status
The Study Will Consist of Taking Some Samples of Crevicular Fluid (the Fluid Found in the Space Between the Gums and the Roots of the Teeth) to Assess a Particular Protein (LL-37) That Seems to be Related to the Immune Response Against Periodontal Disease (Gum Disease)	NCT04404335	Universidad Rey Juan Carlos	Periodontal Diseases Periodontitis	Not yet recruiting (2020)
Liver-enriched Antimicrobial Peptide 2	NCT04043065	University Hospital, Gentofte, Copenhagen	Type 2 Diabetes	Completed (2019)
Role of Antimicrobial Peptides in Host Defense Against Vaccinia Virus (ADVN AMP01)	NCT00407069	National Institute of Allergy and Infectious Diseases (NIAID)	Atopic Dermatitis	Completed (2018)
Characterization of Cutaneous Microbiota in the Psoriasis Pathogenesis (MICROBIOTA)	NCT03475914	Istituto Ortopedico Galeazzi	Psoriasis	Completed (2018)
Antimicrobial Peptides in Periodontitis (PAROPAM)	NCT02793453	CHU de Reims	Periodontal Disease: Chronic Periodontitis	Completed (2017)
Targeted Microbiome Transplant in Atopic Dermatitis	NCT03151148	National Institute of Allergy and Infectious Diseases (NIAID)	Atopic Dermatitis (AD)	Completed (2017)
Vitamin D in Ventilated ICU Patients (R21 HL-110044)	NCT01372995	Emory University	Respiratory Failure	Completed (2017)
Intratumoral Injections of LL37 for Melanoma	NCT02225366	M.D. Anderson Cancer Center	Melanoma	Active not recruiting (2017)
Analysis of Response of Subjects With Atopic Dermatitis or Psoriasis to Oral Vitamin D3	NCT00789880	National Institute of Allergy and Infectious Diseases (NIAID)	Atopic Dermatitis	Analysis of Response of Subjects With Atopic Dermatitis or Psoriasis to Oral Vitamin D3 (2017)
The Estrogen Impact on Overactive Bladder Syndrome: Female Pelvic Floor Microbiomes and Antimicrobial Peptides	NCT02524769	Loyola University	Overactive Bladder	Completed (2015)
PICS: Subtitle Cardiac Dysfunction in Older Sepsis Survivors (PICS)	NCT02276417	University of Florida	Sepsis	Recruiting (2014)
PNEUMOCELL—Conjugated Pneumococcal Vaccination in Patients With Immunoglobulin G-deficiency (PNEUMOCELL)	NCT01847781	Karolinska University Hospital	IgG Deficiency	Completed (2013)
Effects of Vitamin D and Omega-3 Fatty Acids on Infectious Diseases and hCAP18 (VITAL Infection)	NCT01758081	Brigham and Women's Hospital	Infections Human Cathelicidin Antimicrobial Peptide (hCAP-18)	Active, not recruiting (2013)
Immune Reconstitution in HIV Disease (IREHIV)	NCT01702974	Karolinska Institute	HIV Infection	Completed (2012)
Clinical Trial of Phenylbutyrate and Vitamin D in Tuberculosis (TB)	NCT01580007	International Centre for Diarrhoeal Disease Research, Bangladesh	Pulmonary Tuberculosis	Completed (2012)
Immune Reconstitution in Tuberculosis Disease (IRETB)	NCT01698476	Karolinska Institute	Pulmonary Tuberculosis (TB)	Completed (2012)
Effect of Pimecrolimus Cream on Cathelicidin Levels in Subjects With Eczema	NCT00946478	University of California, San Diego	Atopic Dermatitis	Completed (2009)
Effects of Vitamin D Supplementation on Lung Function in an Acute Pulmonary Exacerbation of Cystic Fibrosis	NCT00788138	Emory University	Cystic Fibrosis	Completed (2008)
Therapeutic Induction of Endogenous Antibiotics	NCT00800930	International Centre for Diarrhoeal Disease Research, Bangladesh	Shigellosis	Completed (2008)
Effects of Pimecrolimus on Skin Biopsy Ex-plants From Patients With Atopic Dermatitis	NCT00379678	National Jewish Health	Atopic Dermatitis	Completed (2006)

## Novel delivery approaches for AMPs

There is a higher occurrence of bacterial infection with the development of the bacterial resistance to the conventional antibiotics, which was thought to be solved by the generation of new antibiotics or the development of AMPs. AMPs are of natural origin, which is effective in combating antimicrobial resistance in the place of conventional antibiotics. Nonetheless, development of these newer antibiotics (AMPs) restricts its use due to various problems such as undesirable or nonspecific interactions, proteolytic degradation, and cytotoxicity with limited in vivo activity as well as the stability and selectivity which make the AMPs inefficient to reach the target and exert its action.

**Fig. 3:**
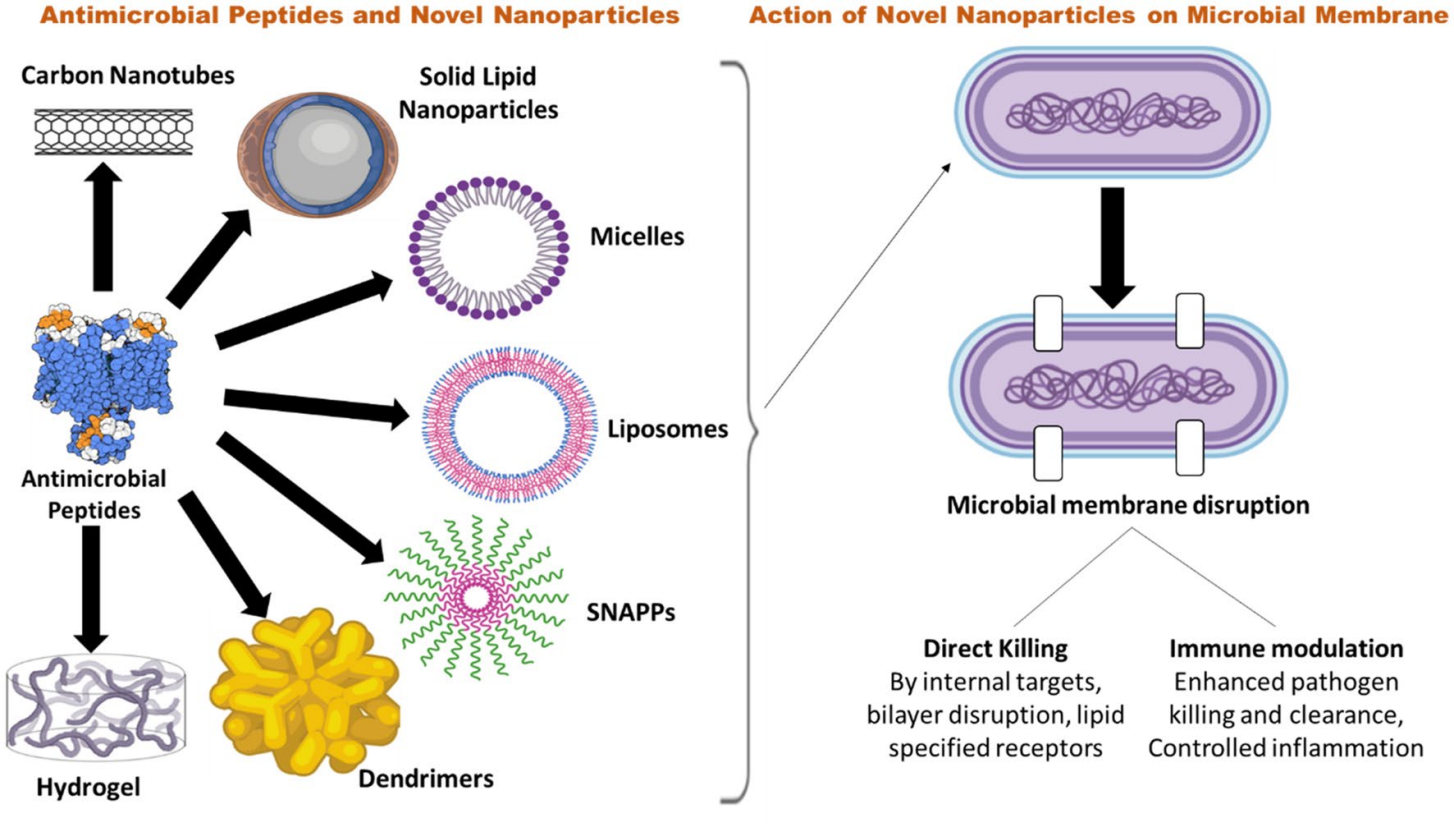
Formulation strategies of Antimicrobial peptides (Martin-Serrano et al. 2019)

Researchers have attempted to develop novel formulation systems for delivering the AMPs, which may help in avoiding the problems associated with delivering AMPs alone. Thus, encapsulating the AMPs into different nanocarriers may provide the direct application for targeting the AMPs in alternate ways. Various scientists have worked on different nanocarriers and successfully encapsulated the AMPs in it and targeted for the AMR therapy such as novel polymeric and lipidic nanoparticles, carbon nanotubes, micelles, liposomes and cubosomes, polymersomes, microspheres, dendrimers, nanocapsules, and other colloidal delivery systems (size up to a few hundred nanometers). These nanocarriers play the role of the transporters to deliver the encapsulated AMPs into the cells which are infected or to the intracellular pathogens. The development of nano formulations loaded with AMPs can aid in avoiding poor bioavailability, proteolysis, or susceptibility and toxicity associated with APMs. Additionally, conjugation of the AMPs with functional polymer provides an excellent antimicrobial activity with new functionalities and also reduces the toxicity by improving its selectivity (Sun et al. 2018). Thus, the development of novel nanocarriers and polymer conjugation concept opens new avenues for the translation of AMPs and its formulations from bench to bedside, However, only a few of the AMPs and its formulations have been translated to clinical trials. The detailed description of different nanocarriers used for delivering the AMPs with their targets is given in Table 4. The formulation strategies explored for the antimicrobial peptides are shown in Fig. 3. (Brandelli 2012; Carmona-Ribeiro and Carrasco 2014; Chen et al. 2014; Almaaytah et al. 2017; Shao et al. 2019; Makowski et al. 2019).

**Table 4:** Different formulation approaches for AMPs

Formulation	Peptide	Target	Description	Advantages	Disadvantages	References
Silver Nanoparticles (Ag-NPs)	Polymyxin B (cyclic polycationic lipopeptide) Gramicidin (hydrophobic AMPs) Alamethicin (hydrophobic AMPs)	Gram-negative bacteria and Gram positive bacteria Gram positive bacteria	Synergistic action of conjugating AMPs with Ag-NPs reduces the MIC to 1–2 µg/ml for both types of AMPs when targeted towards respective microorganism	Effective antimicrobial agent, less toxicity, easy to prepare and eco-friendly to living cells	High cost of power, expensive, Long-term maintenance required	Ruden et al. (2009 and Khan Yasmin et al. (2018)
Polymeric structures (Electospun Polylactic co-glycolic acid)	Magainin II (Mag II)	*Escherichia coli* *Staphylococcus aureus*	Covalent immobilization of AMPs over PLGA and electrospun PLGA/gelatin fibres inhibited bacterial adhesion	Excellent mechanical properties, controlled release and degradability, biocompatible, high surface area and porosity	Complex process and limited to specific polymers	Yüksel and Karakeçili (2014 and Mirjalili and Zohoori (2016)
HEMA hydrogels (2-hydroxyethyl methacrylate) Poly(ethylene glycol)-based (PEG) hydrogel	Polymyxin B and vancomycin AMPs HHC_10_	*Pseudomonas aeruginosa* *Staphylococcus aureus and Staphylococcus epidermidis*	Useful for bacterial eradication Bactericidal and stabilized against the proteolytic degradation	Improved bioavailability, mucoadhesive property, controlled and targeted drug delivery, biodegradable and biocompatible	Chances of burst or rapid release, non-specific drug release, low mechanical strength	Cleophas et al. (2014), Malakooti et al. (2015), Ghasemiyeh and Mohammadi-Samani (2019)
Gold Nanodots (Au-NDs)	Surfactin (SFT)	Methicillin Resistant *S. aureus*-wound healing	Reported reduced MIC upto > 80 folds compared to plain SFT. Faster wound healing and good biocompatibility	Extensive antimicrobial activity, smaller in size with higher surface area, easy to penetrate the bacterial cell wall, better biocompatibility and adaptability	Chances of toxicity	Arvizo et al. (2010), and Chen et al. (2015)
Mesoporous Silica Nanoparticles	LL-37 (cationic AMPs)	Membrane interactions, antimicrobial effect	Anionic porous mesoporous particles has higher loading of cationic AMPs protects from protease degradation	Controlled drug loading and sustained release kinetics, less burst release, good stability and biocompatibility, ease of surface modification, biodegradability	In vivo toxicity	Braun et al. (2016)
Liquid crystalline (LC) structure (cubosomes and hexosomes)	AP-114 (hydrophobic AMPs) DPK-060 (hydrophilic AMPs) LL-37	Phase stability of LC structures and antimicrobial effect of AMPs loaded cubosomes and hexosomes	Good biocompatibility and good stability with LL-37 loaded LC structures. Cubosomes loaded with AP-114 and DPK-060 showed reduced MIC whereas LL-37 loaded resulted in loss of broad spectrum antimicrobial activity	High degree of versatility and biocompatibility, ease to prepare and get narrow particle size distribution and sterilizable,	Highly viscosity in nature, difficult to scale-up	Boge et al. (2016), Naveentaj and Muzib (2020)
Antimicrobial peptide dendrimers (AMPDs)	Tryptophan’s (Trp)	Resistant ESBL *Escherichia coli*	Trp terminating dendrimers reported higher antimicrobial potency with MIC levels depending upon the density of positive charge over the AMPDs	Less immunogenic, smaller production cost, membrenolytic effect,	Expensive and involved complex processes for synthesis and chances of non-specific toxicity	(Scorciapino et al. 2017; Siriwardena et al. 2018; Martin-Serrano et al. 2019)

Researchers have also worked on other novel nanomaterials in addition to the nanocarriers mentioned in Table 4, which are less susceptible to developing resistance against the antibiotics. These novel nanomaterials are star peptide polymers and structurally nanoengineered antimicrobial peptide polymers (SNAPPs). The star-shaped polymers are useful in killing bacteria, which has been proven by in vitro and in vivo studies. In addition, they are less toxic, and it acts through multiple pathways, which may not be possible with conventional antibiotics, thus making this nanocarrier more popular than the conventional nanocarriers (Australasian Science Magazine 2016).

### Star polymers: a ray of hope

The novel delivery system approach has evolved enormously using different polymeric structures to enhance the stability, biocompatibility, and therapeutic efficacy of the drugs. Targeting moieties, hydrophobic, and hydrophilic polymeric carriers, nanoparticles are some of the significant approaches undertaken to improve drug delivery. Star polymers are one such type of delivery system which has gained importance in the field of biomedical applications, starting from gene delivery to antibacterial therapy. Unlike dendrimers, star polymers form a simpler structure which consists of linear arms (unlike dendrimers which consist of branched arms). Certain characteristics like the introduction of functional groups, lower solution viscosity (simpler structure), and biocompatibility have gained an interest in the area of biomedical research (Table 5). The multifunctional core with at least three macromolecular chains representing a star-shaped polymeric structure can attach to a targeting moiety to perform cell-specific targeting. There are three basic synthesis strategies for star polymers; first is the "core first" strategy where arms are coupled with the central multifunctional core. Second is the "coupling-onto" where conjugation reaction between a functional group and the preformed linear arm leads to a comb-shaped like structure. At last, comes the "arm-first" strategy in which covalent interactions are the factor behind attaching the arms with the core using crosslinking agents (Llewelyn et al. 2017; Schuetz et al. 2018).

**Table 5:** Ideal characteristics of Star Polymers for enhanced therapeutic efficacy with certain examples (Llewelyn et al. 2017; Schuetz et al. 2018)

Ideal characteristics	Polymer	Improvement strategy
Well defined structure ATRP^a^ RAFT^b^ Nitroxide-mediated Living anionic/cationic Ring-opening metathesis ROP^c^	β-Cyclodextrin (initiator core)	Controlled molecular weight Low dispersity
Functionality	(a) γ-Cyclodextrin (cationic star polymer) Conjugated with Folic acid residue via a disulfide bond (b) Cell-penetrating peptides (TAT, RGD, GRGDS) (c) Ag, furanone, quaternary ammonium salts groups	(a) Improved gene delivery in cells overexpressing FA receptor (b) Cell adhesion (adhesives like Polyethylene oxide), rapid internalization (c) Long-lasting antibacterial functionality
Stimuli-responsive Enzymatic Redox potential Light pH Temperature	Block copolymers conjugated with pH-sensitive hydrazone moiety. API is DOX^d^	Tumor targeting
Biocompatible	(a) β-Cyclodextrin core (b) Star PLA-Heparin	(a) Temperature responsive hydrogel (b) Hydrophilicity
Biostability and biodegradability	(a) Furanone containing dental cement (b) PLA, PCL, Cyclodextrin	(a) Resistant to light, antibacterial (b) Biodegradable arms and multifunctional core

^a^Atom Transfer Radical Polymerization

^b^Reversible Addition-Fragmentation Chain Transfer polymerization

^c^Ring-opening polymerization

^d^Doxorubicin

Gaining the focus on resistant bacteria (ESKAPE bugs), incorporation of the antibiotic agent to these linear star polymers has resulted in a massive improvement in the antibacterial therapy. This includes attaching either the AMPs or antibacterial groups, which are polycationic, for e.g., poly(2-dimethylaminoethyl methacrylate), PDMAEMA based star polymers are susceptible to *E. coli* (99% in 2 h, MIC-less than 250 µg/ml) (Llewelyn et al. 2017; Schuetz et al. 2018). Studies related to the fact that the introduction of AMPs in these star polymers has proven that this process augments the characteristics of better encapsulation and compartmentalized functionalities of these star polymers, thus giving birth to the concept of stereospecific functionalized stars. Ring-opening polymerization (ROP) technique was adopted for the production of such star polymers. Stereospecific stars, also known as core-cross linked stars (CCS) were synthesized by ROP) of amino acid poly (ε-Z-L-lysine) N-carboxy anhydride (NCA), serving as the arm or the macromolecular initiator, followed by the addition of the cross-linking agent poly (L-cystine). Deprotection of the arms increased the water solubility of the CCS, which further increased the biocompatibility of the stars (Engler et al. 2011; Sulistio et al. 2011a; Wu et al. 2015; Huang et al. 2017).

### Nanostructured antimicrobial peptide polymers

Colistin was the first antimicrobial agent preferred for the resistant Gram-negative bacteria *Acinetobacter baumannii*. However, due to the presence of an extra outer membrane layer and an additional defense mechanism of lipopolysaccharide, these ESKAPE bugs are becoming more and more dangerous and impossible to infiltrate. As mentioned earlier, AMPs incorporated as antimicrobial agents either as polycationic functional groups or amino acid sequences are found to be effective against these resistant microbes via electrostatic interactions but comes up with the adverse effect of toxicity (Sulistio et al. 2011b; Lam et al. 2014, 2016). Thus, exploring the strategy of nanostructured polymeric peptides or SNAPPs have shown excellent activity against both ESKAPE bugs as well as Colistin and MDR *A. baumannii* (CMDR). A mechanism involving outer membrane destabilization, initiation of an apoptotic cell death pathway, and disruption of ion movement across the cell membrane has established the antimicrobial efficacy of SNAPPs. In vitro antimicrobial tests indicated that even after 600 generations of *S. aureus* multiplication, any wild mutations were not observed even in the presence of sub-micron levels of SNAPPs prototype (S16) (Limmathurotsakul et al. 2019) revealing that these SNAPPs did not develop resistance.

Stereospecific structures of functional AMPs are commercially developed by using ROP-NCA (ring-opening polymerization N-carboxy anhydride) technique. In a recent study, SNAPPs were prepared using ROP-NCA which utilized lysine (cationic) and valine (hydrophobic) as amino acid residues. PAMAM dendritic arms using lysine to valine ratio of 2:1 to increase the water solubility of the structure were synthesized. The structures containing homolysine residues had a higher minimum bactericidal concentration (MBC) against *E. coli*. Furthermore, the antibacterial efficacy was found to be not species-specific. Localization of the charges owing to the nanostructure increases the bacterially induced peptide aggregation and thus increases the efficacy of the AMPs, which are formulated as SNAPPs. Unlike host defense peptides which only show bacterial pathway to directly prevent the activity of the ESKAPE bugs, SNAPPs show both bacterial as well as indirect pathway of immunizing the mammalian cells against CMDR and ESKAPE pathogens. The above mentioned indirect pathway is exhibited by increasing the neutrophil infiltration mechanism (O’Neill 2014; Lam et al. 2016).

Other strategies to develop AMPs are NCA-ROP techniques for utilizing alpha-amino acids. MRSA, *P. aeruginosa*, *Serratia marcescens*, and *C. albicans* (Devadas et al. 2019) were found to be highly susceptible at the lowest MIC when AMPs comprising of lysine (hydrophilic moiety), phenylalanine and leucine as the hydrophobic moiety in the ratio of 10:7.5:7.5 and lysine, phenylalanine in the ratio of 10:15 were used (Zhou et al. 2010; Raju 2011). Cationic polymers are preferred as they exhibit electrostatic interactions with the bacterial surface. Examples of certain synthesized cationic polymers include polyethyleneimines, polymethacrylates, polyarylamides, and protonated polyesters. Further, the development of polypeptide libraries by varying the carbon chain length of the side group functionalities gave an overall idea that they were effective against a broader spectrum of Gram-positive and Gram-negative bacterial populations and also prevent biofilm formation especially against *E. coli* and *S. aureus* (World Health Organization 2001; Engler et al. 2011).

## Conclusion

A dearth of the antimicrobial agents has led to a major concern regarding infectious disease control. ESKAPE bugs have become self-sufficient in destroying every other antimicrobial delivery strategy. Numerous novel drug delivery systems that mimic the natural bacteriolytic action of peptides have been studied involving AMPs, incorporating those peptides as a nano-formulation and introduction into the star polymers. SNAPPs which describe the ultimate architecture of the star polymers have shown a promising future in combating AMR because of the additional apoptotic mechanism which is switched on by these SNAPPs once it gains access to the bacterial cell. Synthesis techniques of the AMPs have been in the limits of using ROP technique. However, extensive research in this field for the synthesis of AMPs to yield a cost-effective and reproducible outcome is very necessary. Modulation of the functionalities on the surface of the star polymers to check for a range of the therapeutic activity may be the futuristic goal for the upcoming research.
